# Prevalence, clinical characteristics, and outcome of pleural effusions in ovarian cancer

**DOI:** 10.1515/pp-2020-0152

**Published:** 2021-03-24

**Authors:** José M. Porcel, Paola Murata, Laura Porcel, Silvia Bielsa, Marina Pardina, Antonieta Salud

**Affiliations:** Pleural Medicine Unit, Department of Internal Medicine, Hospital Universitari Arnau de Vilanova, IRBLleida, University of Lleida, Lleida, Spain; Department of Medical Oncology, Hospital Universitari Arnau de Vilanova, IRBLleida, Lleida, Spain; Department of Internal Medicine, Hospital Universitario Principe de Asturias, Alcalá de Henares, Madrid, Spain; Department of Radiology, Hospital Universitari Arnau de Vilanova, IRBLleida, Lleida, Spain

**Keywords:** indwelling pleural catheter, ovarian cancer, pleural effusion, pleurodesis, survival

## Abstract

**Objectives:**

The prevalence, clinical characteristics and prognosis of pleural effusions (PEs) associated with ovarian cancer (OC) have seldom been addressed systematically, as in the current investigation.

**Methods:**

All records of consecutive women with a newly diagnosed OC in our institution over a 13-year period were retrospectively reviewed. Features of PEs on CT scans, pleural fluid analyses, need for definitive therapy of PEs, and the influence of PEs on the overall survival (OS) and progression-free survival (PFS) were evaluated.

**Results:**

PEs were observed in 81 (43%) of 189 women with OC, either at presentation of cancer (55 patients) or during the course of the disease (26 patients). The causes of PEs were malignancy (55.5%), unknown (37%), or surgery-related (7.4%). The sensitivity of the cytologic diagnosis of malignant PEs was 79.1%. Sixty percent of malignant PEs required pleurodesis or indwelling pleural catheters for symptomatic relief. The presence of ascites strongly predicted PE development (odds ratio 43.2). Women with PEs fared much worse compared with those without PEs, in terms of OS (26.7 vs. 90.4 months), PFS (9.8 vs. 55.3 months) and tumor recurrences (86.4 vs. 43%). In multivariate analyses, PE remained as a relevant independent variable associated with poor outcome (hazard ratio 9.73 for OS, and 3.87 for PFS). Notably, PEs small enough to preclude tapping, and thus of unknown origin, had a similar bad prognosis as malignant PEs.

**Conclusions:**

OC patients with PEs experience decreased survival, including those with trace effusions not amenable to tapping.

## Introduction

Ovarian cancer (OC) encompasses a heterogeneous group of malignancies, over 90% of which are of epithelial origin [1]. Fewer than half of patients with OC survive beyond five years after diagnosis, partly because more than 70% are discovered in an advanced disease stage [1]. OC was the sixth leading cause of 840 tapped malignant pleural effusions (MPEs), after lung, breast, unknown primary, hematological, and gastrointestinal tumors [2]. However, it ranks third if only women are being considered [3]. OC often initially manifests with a pleural effusion (PE). In a retrospective analysis of 32 consecutive OC women with MPE, the effusion was among the presenting symptoms of cancer in 24 (75%) cases [4].

Few studies have addressed the prognostic significance of PEs in the context of specific primary malignancies. For example, PEs, including those too small to be aspirated, have been found to be an independent predictor of poor survival in lung cancer [5] and diffuse large B-cell lymphoma [6]. The only two such studies conducted in OC provided a partial view of the problem because they included a very select population. One analyzed a small number of patients with cytology-proven MPEs who underwent optimal abdominopelvic cytoreductive surgery [7], while the other focused on PEs detected before a primary debulking surgery for advanced cancer grades [8]. In the current investigation we aimed, for the first time, to systematically analyze the etiology, clinical characteristics, and outcomes of PEs at any OC stage, as well as the influence they may have on overall survival (OS) and progression-free survival (PFS).

## Materials and methods

### Study design

We retrospectively reviewed all consecutive patients who were newly diagnosed with OC from January 2007 to December 2019 at our academic teaching hospital. The study was approved by the local Ethics Committee (CEIC No. 1965).

### Data collection

Electronic medical records were queried for the following variables: age at diagnosis of cancer, serum concentrations of CA125 at tumor detection, tumor histology, identification of potential OC-susceptibility genes, presence and characteristics of PE (side and volume according to a previously reported formula) [9] on computed tomography (CT) at any time from the diagnosis, identification of pleural thickening ≥3 mm and/or nodularity, ascites, or any metastatic site on CT imaging, biochemical and cytological pleural fluid (PF) data when available, Easter Cooperative Oncology Group (ECOG) performance score and International Federation of Gynecology and Obstetrics (FIGO) staging system at the first encounter, treatments received (optimal or suboptimal cytoreductive surgery, chemotherapy, antiangiogenesis therapy, poly ADP-ribose polymerase [PARP] inhibitors), recurrence of disease on the basis of radiological criteria, need for pleural interventional procedures (therapeutic aspirations, pleurodesis, tunneled pleural catheters), OS and PFS. For bilateral PEs, the effusion with the greater size was recorded.

### Diagnostic criteria for pleural effusions

A PE was categorized as malignant if malignant cells were detected upon cytological examination of PF (whether smears or cell blocks) or biopsy specimens. A diagnosis of probable MPE was based on the demonstration of a cytology-negative exudate, after reasonably ruling out benign causes of fluid accumulation. Other etiologies of PEs were defined using well-established clinical criteria [10]. Untapped PEs were classified as being of uncertain origin, unless clearly ascribed to a postoperative complication (postsurgical PEs). Interpretation of chest and abdominal CT imaging was done by an expert radiologist who was blinded to clinical data, other than that the patient had OC.

### Statistical analysis

Continuous and categorical variables were expressed as medians (quartiles 25th and 75th) and percentages, respectively. For between-group comparisons, either the Chi-square, Kruskal–Wallis or Mann–Whitney U tests were used, whichever was appropriate. A logistic regression analysis was performed to determine predictors of PE development. OS was calculated from date of OC diagnosis until death or last follow-up. PFS was defined as the time from initiation of any active oncologic treatment to the occurrence of progressive disease, relapse, or death. The Kaplan–Meier method and Cox proportional hazard regression model were used for calculation of survival over time, while survival differences were analyzed using the log-rank test. The optimal cutoff values for PE size (expressed in mL) and preoperative CA 125 (U/mL) were determined by receiver operating characteristic curve analysis, looking for a specificity of 85% to predict death or cancer recurrence. A p value <0.05 was considered statistically significant in all analyses. Calculations were done using SPSS version 24.0 statistical software.

## Results

### Study population

A total of 189 women with a median age of 63 years (range, 28–93 years) were diagnosed with OC during the 13-year study period. Twenty-six (26%) of 99 tested patients carried pathogenic mutational variants, mostly BRCA1 and/or BRCA2 (17%). Histological types, FIGO disease stages and ECOG performance status at diagnosis, and therapeutic approaches are shown in Table 1. OC was located on the right side in 25% of the cases, on the left in 30%, and bilaterally in 45%. Standardized surgery was attempted in 157 (83%) women, though optimal abdominopelvic cytoreduction was achievable in 78 (50%).

**Table 1:** Baseline characteristics of OC patients.

Characteristics	All patients (n=189)	OC with pleural effusion (n=81)	OC without pleural effusion (n=108)	p-Value^a^
Age, years	63 (52–71)	65 (58–77)	60 (48–69)	<0.001
Carriers of genetic mutations	26/99 (26.3%)	8/31 (25.8%)	18/68 (26.5%)	0.944
CA125 in serum at diagnosis, U/mL^b^	383 (77–1,224)	785 (306–3,158)	144 (41–598)	<0.001
Pleural nodularity and/or thickening on CT^c^	17 (9%)	17 (21%)	0 (0%)	<0.001
ECOG performance status				
0	72/187 (38.5%)	23 (28%)	49/106 (46%)	
1	89/187 (47.5%)	44 (54%)	45/106 (43%)	0.088
2	23/187 (12.3%)	12 (15%)	11/106 (10%)	
3	3/187 (1.6%)	2 (3%)	1/106 (1%)	
FIGO stage				
I	30 (16%)	4 (5%)	26 (24%)^d^	
II	6 (3%)	2 (2.5%)	4 (4%)	<0.001
III	97 (51%)	32 (39.5%)	65 (60%)	
IV	56 (30%)	43 (53%)^d^	13 (12%)	
Histological type				
High-grade serous carcinoma	132 (70%)	60 (74%)	72 (66.7%)	
Low-grade serous carcinoma	4 (2%)	0 (0%)	4 (3.7%)^d^	
Endometrioid	14 (7.4%)	3 (3.7%)	11 (10.2%)^d^	0.012
Clear cell	12 (6.3%)	3 (3.7%)	9 (8.3%)	
Mucinous	3 (1.6%)	0 (0%)	3 (2.8%)	
Non-differentiated	18 (9.5%)	13 (16%)^d^	5 (4.6%)	
Others	6 (3.2%)	2 (2.5%)	4 (3.7%)	
Metastatic locations				
Peritoneal	158 (83.6%)	79 (97.5%)	79 (73%)	<0.001
Ascites	128 (67.7%)	78 (96.3%)	50 (46.3%)	<0.001
Lymph nodes	52 (27.5%)	32 (39.5%)	20 (18.5%)	0.001
Liver	28 (14.8%)	16 (19.8%)	12 (11%)	0.098
Lung	12 (6.3%)	3 (3.7%)	9 (8.3%)	0.196
Bone	8 (4.2%)	8 (9.9%)	0 (0%)	0.001
Brain	6 (3%)	5 (6%)	1 (0.9%)	0.042
Soft tissue	6 (3%)	5 (6%)	1 (0.9%)	0.042
Spleen	3 (1.6%)	2 (2.5%)	1 (0.9%)	0.401
Treatments				
Surgery	157 (83%)	59 (73%)	98 (91%)	0.001
Optimal cytoreductive surgery	78 (49.7%)	16 (27.1%)	62 (63.3%)	0.001
Adjuvant chemotherapy	139/186 (75%)	49/80 (61%)	90/106 (85%)	<0.001
Neoadjuvant chemotherapy	68 (36%)	41 (51%)	27 (25%)	<0.001
Angiogenesis inhibitors	47 (25%)	23 (28.4%)	24 (22%)	0.331
PARP inhibitors	11 (6%)	2 (2.5%)	9 (8.3%)	0.088
Palliative care	10 (5.3%)	9 (11.1%)	1 (0.9%)	0.002
Recurrent disease	95/161 (59%)	51/59 (86.4%)	44/102 (43%)	<0.001
Deaths	112 (59.2%)	74 (91.3%)	38 (35%)	<0.001

^a^p-Value for the comparison of OC with and without pleural effusions. ^b^Data on serum CA125 was available in 160 patients (67 with and 93 without pleural effusions). ^c^The etiologies of pleural effusions associated with pleural nodularity and/or thickening were malignancy (n=12) and unknown (n=5). ^d^Significantly superior than the comparator group. CT, computed tomography; ECOG, Easter Cooperative Oncology Group; FIGO, Federation of Gynecology and Obstetrics; OC, ovarian cancer.

### Characteristics of pleural effusions

At presentation of OC, 55 (29%, 95%CI 23–36%) patients showed PEs on CT images. Twenty-six additional women developed PEs during the course of the disease following a median of 5.2 months (six days–30.6 months) from tumor diagnosis. Thus, the overall prevalence of PEs was 42.8% (81 of 189 patients, 95%CI 36–50%). Some differential features of OC women with and without PEs are displayed in Table 1. Notably, ascites was much more prevalent in those with PEs (96.3 vs. 46.3%, p<0.001). Pleural nodularity or thickening on CT scans, a radiological sign suggestive of malignancy, was invariably associated with the presence of PEs, in particular MPEs and, less often, PEs of uncertain cause. Moreover, women with PEs developed recurrent OC more frequently (86.4 vs. 43.1%, p<0.001) and in a shorter time period from cancer diagnosis (12.6 vs. 20.8 months, p=0.003) than those without PEs.

In a multivariate analysis, which included the statistically significant variables of the univariate analysis (Table 1), independent risk factors for the occurrence of PEs in OC patients were the existence of ascites (odds ratio [OR] 43.2, 95%CI 5.5–337.8), extra-pleural recurrences (OR 4.4, 95%CI 1.6–12.3) and peritoneal metastases (OR 3, 95%CI 1.2–7.2).

PEs were bilateral in 43 cases (53%), right-sided in 21 (26%) and left-sided in 17 (21%). The etiologies of the 81 PEs were definitely malignant (36, 44.4%), probable malignant (9, 11.1%), uncertain (30, 37%) and benign (six postsurgery, 7.4%). The six PEs in women with FIGO stages I and II were related to surgery (n=2), or undefined causes (n=4). The median volume of PEs was significantly higher in the malignant group (400 mL [200–1,200] vs. 100 mL [65–225], p<0.001). None of the subjects with PEs of uncertain cause underwent thoracentesis, usually because of the small quantity of fluid (median 100 mL, quartiles 50–500). PF was eventually analyzed in 45 (55.5%) patients. All MPEs were exudates, with a lymphocyte-predominance in 93% of the cases, and the overall cytologic yield for the examination of two separate fluid specimens (smears and/or cell blocks) was 79.1% (72.1% if only one specimen had been submitted). Of the 45 subjects with MPEs, 30 (66.7%) required one or more palliative pleural procedures (18 therapeutic thoracentesis, 14 bedside pleurodesis, and 13 indwelling pleural catheters).

### Survival

**Figure 1:**
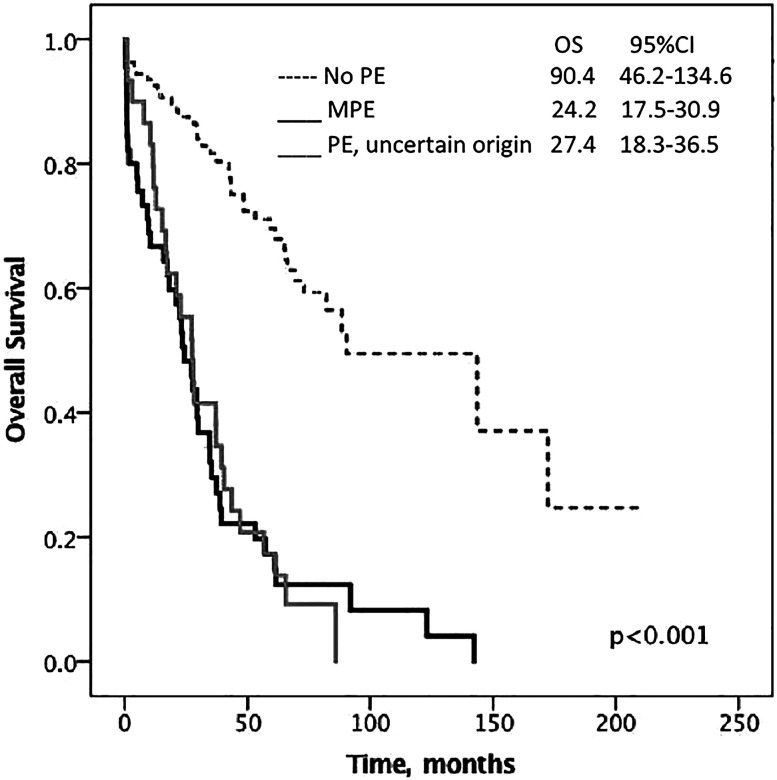
Median overall survival of ovarian cancer patients according to the pleural effusion status. OS, overall survival; MPE, malignant pleural effusion; PE, pleural effusion.

At the end of the study, 112 (59%) patients had died and the median follow-up time for the remaining 77 was 59.1 (25.8–80.1) months. Median OS for the entire cohort was 48.5 months (95%CI 33.8–63.2). Women with PEs survived less than those without PEs (26.7 months, 95%CI 22–31.3 vs. 90.4 months, 95%CI 46.2–134.6; p<0.001). Specifically, OS was significantly lower in the subgroup of patients with either MPEs (24.2 months, 95%CI 17.5–30.9) or PEs of uncertain origin (27.4 months, 95%CI 18.3–36.5) as compared to the non-PE group (p<0.001) (Figure 1). Although there was a trend toward a better OS in women whose PEs appeared during the disease course (34.5 months, 95%CI 25.4–43.5) in contrast to those with PEs at the time of OC diagnosis (20.8 months, 95%CI 13.9–27.7), it did not reach statistical significance (p=0.296).

A multivariate analysis using the Cox regression model was performed, after entering the following variables as potential prognostic factors: age, serum CA 125 ≥ 1,200 U/mL at diagnosis, FIGO stage, ECOG, OC histological type, metastatic pattern, presence of PEs, volume of PEs >250 mL, pleural nodularity and/or thickening on CT scans, ascites, as well as surgical therapy and its results (optimal or suboptimal cytoreduction). As shown in Table 2, the presence of PE was one of the strongest predictors of decreased survival (hazard ratio [HR] 9.73). Exclusion of the six postsurgical PEs for the calculations did not change results (data not shown).

**Table 2:** Cox proportional hazard models assessing overall survival of OC patients.

Variable	Adjusted HR (95%CI)	p-Value
Presence of PE	9.73 (4.19–22.6)	<0.001
No surgical treatment	7.46 (4.19–13.30)	<0.001
Peritoneal metastases	4.77 (1.49–15.27)	0.009
PE volume >250 mL	2.12 (1.31–3.43)	0.002
Suboptimal cytoreductive surgery	1.97 (1.20–3.24)	<0.001

CI, confidence interval; HR, hazard ratio; OC, ovarian cancer; PE, pleural effusion.

**Figure 2:**
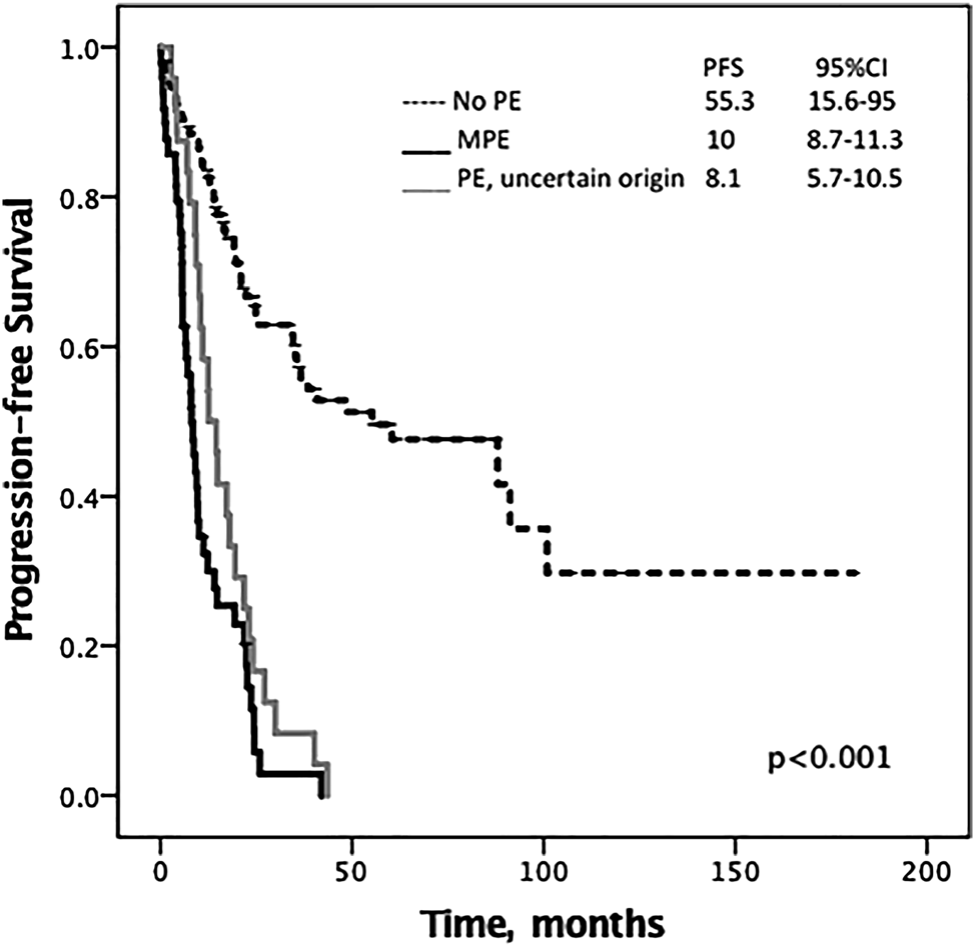
Median progression-free survival of ovarian cancer patients stratified for pleural effusion status. MPE, malignant pleural effusion; PE, pleural effusion; PFS, progression-free survival.

Median PFS of the study population was 21.9 months (95%CI 18.3–25.6). It was lower in the PE cohort (9.8 months, 95%CI 8.4–11.6) than in the non-PE group (55.3 months, 95%CI 15.6–95; p<0.001) (Figure 2). MPEs and PEs of uncertain etiology exhibited similar PFS (10 months, 95%CI 8.7–11.3 vs. 8.1, 95%CI 5.7–10.5; p=0.593) (Figure 2). There was also a nonsignificant tendency toward a better PFS when PEs developed during follow-up rather than being discovered at the time of OC diagnosis (12.6 months, 95%CI 8–17.2 vs. 8.1 months, 95%CI 5.5–10.6; p=0.081).

The multivariate Cox regression model showed that, for the same variables as those previously mentioned for the OS statistical model, PEs were included among the most significant predictors of a poor PFS (HR 3.87) (Table 3).

**Table 3:** Cox proportional hazard models assessing progression-free survival of OC patients.

Variable	Adjusted HR (95%CI)	p-Value
Lung metastases	6.27 (3.17–12.4)	<0.001
No surgical treatment	5.10 (2.71–9.60)	<0.001
Presence of PE	3.87 (2.52–5.96)	<0.001
Suboptimal cytoreductive surgery	2.81 (1.79–4.41)	<0.001

CI, confidence interval; HR, hazard ratio; OC, ovarian cancer; PE, pleural effusion.

## Discussion

To our knowledge, this study represents the first simultaneous evaluation of the prevalence, clinical and PF features, and prognosis of PEs in women with OC. PEs occurred in 43% of the cases, using CT as the reference standard, and independently contributed to mortality and PFS.

Most OC were diagnosed at FIGO stages III (51%) and IV (30%). Since PE with positive cytology defines FIGO stage IVA, reasonably the majority of detected PEs fell into the advanced stages III (39.5%) and IV (53%). The overall prevalence of PEs in OC (43%) was quite similar to that reported for other primary tumors that metastasize to the pleura, such as lung cancer (40%) [5] or diffuse large B-cell lymphoma (30%) [6]. Pleural nodularity or thickening characterized 27% of MPEs in OC women, which is somewhat lower than the 43% general prevalence of these specific radiological signs in pleural malignancies [11]. However, the potential malignant nature of PEs that were classified as being of uncertain origin cannot be excluded (16.7% of which exhibited pleural nodules and/or thickening). Also, in the context of an OC, it was found that the larger the accompanying PE, the more likelihood of being malignant. This is not surprising, considering that malignancy accounts for more than half of large or massive PEs [12]. In a study of 16 OC patients with MPEs and 22 with nonmalignant PEs, moderate to large amounts of PF (81.3 vs. 9.1%) and pleural nodules (50 vs. 0%), along with supradiaphragmatic lymph node enlargement (75 vs. 9.1%) predominated in the former group and, therefore, these CT signs increased the probability for a PE to be malignant [13].

The yield of PF cytological examinations seems to be particularly high in MPEs secondary to OC as compared to other tumor types. While the overall sensitivity of PE cytology for identifying malignancy is around 55% [14], it was found this percentage reached 79% in our cohort of OC women, which is in line with other reports. For example, in four series comprising 27, 31, 38, and 96 MPEs due to OC, the respective PF cytological yields were 83, 84, 94.7, and 78% [15], [16], [17], [18], though no information on the number of fluid specimens processed and the way they were examined (whether smears and/or cell blocks) were provided. Another point was that MPEs in OC were symptomatic enough to require definitive pleural procedures, either bedside pleurodesis or insertion of an indwelling pleural catheter, in 60% of the cases. This percentage compares with that observed in a multicenter cohort of 537 MPEs from a variety of tumor types (OC representing only 5.8% of the cases), where 288 (53.6%) eventually needed to be treated with pleurodesis and/or indwelling pleural catheters [19].

The mechanism of metastatic pleural involvement in OC is speculative and probably includes one or more of the following: a) invasion from an affected contiguous diaphragm, b) migration of tumor cells from the peritoneal cavity to the pleural space through small diaphragmatic defects, and c) hematogenous spread. The relevance of the first two potential ways of cancer dissemination was supported by the finding of ascites and peritoneal metastases (which encompasses peritoneal diaphragmatic implants) as independent predictors of PE development.

Our survival analysis showed that women with PEs (particularly PEs of at least 250 mL), had worse OS and PFS than those without PEs after adjustment for confounders. A pair of prior studies cast a sobering light on the prognostic impact of PEs in OC women. In an earlier retrospective study, 21 OC patients with stage IVA (due to MPEs), who underwent tumor debulking to less than 1 cm of residual disease followed by platinum-based chemotherapy, had a shorter time to recurrence (12 vs. 21 months, p=0.04) and decreased OS (30 vs. 58 months, p=0.016) compared to 76 similarly treated patients with stage IIIC [7]. However, CT scans were not used for staging and, therefore, the existence of undetected intrathoracic disease could not be excluded. In a second study from the same institution, the preoperative CT images of 172 stage III and 31 stage IV OC women undergoing primary cytoreduction were retrospectively evaluated [8]. The prevalence of PEs at diagnosis (62 patients, 30.5%) was almost identical to that observed in our study. However, only 19 (30%) were tapped, of which 16 were deemed to be malignant. In both stages III and IV the presence of PEs occupying one-third or more of the hemithorax was independently associated with worse OS in a multivariate analysis (HR=2.27) [8].

A novel finding of our study is that even trace effusions not amenable to tapping confer a survival disadvantage, with a differential life expectancy of 63 months as compared to patients without PEs. It can be hypothesized that many of these minimal effusions are actually MPEs which go unnoticed due to the difficulty of performing conventional diagnostic procedures. It should be stressed that an accurate identification of the presence and extent of pleural involvement, though paramount for determining prognosis and selecting appropriate treatment, is difficult by noninvasive means. The true pleural status revealed during video-assisted thoracoscopic surgery (VATS) do not always correlate with the CT features considered suggestive of pleural malignancy (i.e., thickening and/or nodularity) [20], nor with the PF cytology results [18], [19], [20], [21]. For instance, in a series of 44 advanced OC patients who underwent VATS, 10 and 16 had pleural nodules and pleural thickening suspicious of malignancy on preoperative CT scans, respectively [20]. However, thoracoscopic biopsies did not confirm pleural tumor invasion in two and six of these cases. In a recent study, VATS was performed on 100 women with advanced OC and PEs occupying one-third or more of the pleural cavity [18]. Macroscopic pleural disease was identified in 70% and was independently associated with elevated death risk. Interestingly, 29% of patients with negative PF cytology had gross macroscopic disease at VATS [18]. Conversely, no gross pleural disease is seen during VATS procedures in around 25% of OC patients with positive PF cytology [21]. These data support the indication of VATS in all OC women with moderate to large PEs in order to confirm macroscopic pleural disease that may impact management [18]. Currently, it has not been established whether bulky pleural involvement is present in OC women with small PEs and if VATS could be beneficial in these patients.

Our study has several limitations. First, our cohort was sourced from a single institution and spanned a relatively long-period of time during which therapeutic approaches have varied. Second, the sample size was moderate and the study design retrospective, though there were very little missing data. Third, our reference standard for some MPEs (those labeled as probable MPEs) was imperfect, but widely accepted in medical literature. Finally, 44% of PEs could not be analyzed due to their small volume which, on the other hand, reflects daily clinical practice. Yet demonstration of the poor outcome of these minimal PEs is a relevant fact.

In summary, PEs are frequently encountered in OC women and portend a poor prognosis, even those of such a small size that a diagnostic thoracentesis cannot be performed safely. Nearly two-thirds of MPEs associated with OC need definitive pleural procedures, either pleurodesis or indwelling pleural catheters, for symptomatic control. Whether minimal PEs harbor undiagnosed pleural involvement should be explored in future investigations.
